# From Knowledge to Practice: Evaluating a Targeted Faculty Development Program for Medical Educators Using the Kirkpatrick Framework

**DOI:** 10.1007/s40670-026-02803-7

**Published:** 2026-07-09

**Authors:** Ahdeah Pajoohesh-Ganji, Kirsten Brown, Tayla Beckles, Debra Herrmann

**Affiliations:** 1 Department of Anatomy and Cell Biology, George Washington University School of Medicine and Health Sciences, DC Washington, USA; 2 Center for Faculty Excellence, George Washington University School of Medicine and Health Sciences, DC Washington, USA; 3 Department of Physician Assistant Studies, School of Medicine and Health Sciences, George Washington University, DC Washington, USA

**Keywords:** Faculty development, Kirkpatrick program evaluation, Teaching and learning, 4-Cs framework

## Abstract

Limited Teaching Faculty (LTF) are often asked to teach occasional sessions without formal pedagogical training. To address this gap, we developed the Teaching Essentials Series (TES), a faculty development program tailored to help these educators become more effective teachers. We evaluated TES using a case study approach guided by the Kirkpatrick evaluation framework, utilizing quantitative and qualitative methods. Pre- and post-surveys assessed changes in participants’ knowledge and confidence. In addition, qualitative semi-structured interviews were conducted with program participants from 2023 to 2025. The Kirkpatrick model informed both the development of the interview guide and the interpretation of findings. Quantitative findings indicate that TES is a beneficial program that enhances participants’ knowledge and confidence. Qualitative interviews revealed four central themes: (1) the need for formal pedagogical training, (2) the value of practical learning, shared strategies, and peer connections, (3) enhanced confidence and educational profile, and (4) a shift in teaching perspective and mindset. Participants reported that TES helped them develop practical teaching skills that they could share with peers and leaders, with minimal time commitment. Aligned with the 4-Cs framework (Competence, Career, Context, and Community), the evaluation of the TES program suggests that TES represents a feasible, low-time-commitment model for supporting educators with substantial research and clinical responsibilities. Furthermore, participants noted that TES equipped them with tools they could share with colleagues and leaders, reinforcing the program’s broader impact.

**Supplementary Information:**

The online version contains supplementary material available at https://doi.org/10.1007/s40670-026-02803-7.

## Introduction

Faculty in research-intensive institutes and clinicians in teaching hospitals undergo rigorous training in research, grant writing, patient care, and clinical training. However, the lack of formal pedagogical training compels these faculty members to rely on self-developed teaching methods that are often based on trial and error or on personal experience as learners, rather than on evidence-based educational practices [1–4]. Studies have shown that faculty development is crucial in enhancing teaching effectiveness and student success [5, 6]. In a survey of faculty members, 96% support more formalized training in teaching [7]. Evidence indicates that structured pedagogical training improved faculty confidence, student satisfaction, and course performance [7]. However, this work has focused on higher education broadly, rather than on medical education.

While multiple studies discuss and evaluate faculty development programs to enhance the teaching of clinical educators, very few examine how to improve the teaching of basic science faculty. Skeffe and colleagues developed a faculty development program specifically for pathology faculty whose teaching was a small portion (15%) of their academic work [8]. Although they found that their brief teaching intervention enhanced teaching effectiveness, the program had only eight participants. More recently, Steinert and colleagues found that only 10% of faculty development initiatives target both basic scientists and clinicians. These studies demonstrate the paucity of targeted programming for these faculty groups, informing our decision to design an intervention inclusive of both groups [9].

Many institutions provide faculty development programs, some of which are highly advanced and require a significant time commitment. For instance, Johns Hopkins School of Medicine offers a *Teaching Skills Program* consisting of a 24-session in-person program and a 10-session virtual program [10], both of which meet for 2 to 3.5 h weekly. Harvard Medical School offers the *Training to Teach in Medicine Program* to enhance faculty’s teaching and educational leadership skills [11] It is delivered through two live online workshops held several months apart, with project-mentored work in between. Participants are expected to spend 7–10 hours per week on coursework. Similarly, the George Washington University School of Medicine & Health Sciences (GW SMHS) offers the *Master Teacher Leadership Development Program (MTLDP) *[12], a nine-month, longitudinal, five-course graduate certificate program that meets for 4.5 h weekly. These programs are highly effective in enhancing teaching skills, developing educational leadership, and cultivating collaborative, small-group learning. However, to maintain the quality and impact of the experience at our institution, the MTLDP can only accommodate 12–14 faculty members annually. Expanding enrollment beyond this capacity would require significantly more resources, including additional faculty time and logistical support. Furthermore, such programs require participants to have a substantial teaching load to apply the knowledge effectively. Therefore, this leaves a gap in faculty development programming for individuals without extensive traditional teaching responsibilities, who play a crucial role in medical education.

To address this gap, we developed the “Teaching Essential Series” (TES), a program tailored for faculty who are asked to teach limited, selected sessions within their expertise. We define these individuals as Limited Teaching Faculty (LTF), or those faculty whose overall efforts are more heavily weighted for research (e.g., basic scientists) or clinical care. TES provides these faculty with foundational training in educational concepts and practices. This article presents the findings of a three-year evaluation of the TES program, exploring participants’ perspectives on its value as an essential teaching tool. Additionally, we outline the challenges and opportunities identified during the quality improvement process to help readers implement a similar program at their institution.

## Methods

We chose a case study research design to evaluate TES for several reasons. First, TES is an innovative, unique program implemented at our institution and targeted toward LTF educators with limited training and opportunities to teach [13]. Second, this approach allows for a detailed examination of the TES’s design, delivery, and outcomes, focusing on participants’ experiences, program strengths, and areas for improvement. Lastly, by using surveys and interviews, this design provides a comprehensive, multidimensional perspective on the program, which aligns well with the case study design [14].

This case study was bounded by the TES as implemented at the George Washington University, focusing on three consecutive cohorts offered in the fall semester from 2022 to 2025. The case included faculty, clinicians, and trainees who participated in TES during this period, regardless of whether they completed all sessions or only a subset, reflecting the program’s flexible, voluntary structure. Data were limited to surveys and interviews collected from the participants within the cohorts, ensuring a focused examination of experiences.

### Evaluation Framework

We selected Kirkpatrick’s Model as our framework because it is widely used for evaluating the effectiveness of training programs [15]. Kirkpatrick’s model consists of four levels: reaction, learning, behavior, and results. For our project, the first level measures participants’ immediate reactions to the training, including their satisfaction and engagement with the material. The second level assesses the effectiveness of the training by measuring whether the participants acquired the intended knowledge, skills, and confidence. The third level evaluates how effectively participants applied their newly acquired knowledge in their work environment and whether they could transfer the knowledge from the second level. The fourth level measures the overall impact of training on the organization. To ensure the evaluation was structured and theoretically grounded, the interview questions were aligned with all levels of the Kirkpatrick framework (Supplemental Table 1).

The research questions were: (1) To what extent did the participants find the TES beneficial? This question evaluates participants’ perception of the program’s relevance to their needs, representing the reaction level (i.e., satisfaction); (2) What impact did the program have on the participants’ confidence? This question examines changes in participants’ confidence resulting from the program, reflecting the learning level; (3) What impact did the program have on the way participants teach? This question assesses whether participants have applied their learning to their teaching practices and indicates the behavior level; and (4) To what extent did the participants think the program was applicable across departments? This question addresses participants’ perceptions of the program’s impact on their teaching and/or whether they shared it with others, representing the results level.

### Program Description

The setting for our study was the GW SMHS. TES was officially launched in the fall of 2021; however, a formal evaluation of the program did not occur until 2022. TES consists of five Units covering the following topics: (1) Writing Learning Objectives, (2) Assessments, (3) Instructional Design, (4) Writing Multiple-Choice Questions (MCQs), and (5) Balancing Teaching, Research, and Service. An overview of TES topics, learning objectives, and activities is provided in Supplemental Table 2. Each Unit comprises an asynchronous and a synchronous component. The asynchronous component is an online module that includes multimedia background information and preparatory work shared with participants via an online learning management system. The synchronous component was held via Zoom, a cloud-based videoconferencing service (Zoom Video Communications, Inc., San Jose, California), where the presenters emphasized continued application and engagement with unit content, including a brief review of the asynchronous preparatory work, group discussions, idea sharing, and Q&A. Starting in 2022, TES offered continuing medical education (CME) credit to incentivize clinical educators.

TES was advertised to all full-time and part-time faculty through faculty listservs and shared with leaders of all educational programs. Program registrants were emailed calendar invites with Zoom information and links to asynchronous content. All materials were distributed upon completion of each unit.

### Study Population

Our study population comprises prior TES registrants from 2022 to 2024 (*n* = 425). These individuals, including faculty, trainees, and staff, registered to participate in TES and worked at GW SMHS and associated clinical sites.

### Data Collection

The GW Office of Human Research determined that this project did not meet the regulatory definition of human subjects research and therefore did not require Institutional Review Board oversight (Protocol # NCR213938). Nonetheless, we provided all interview participants with an overview of the project and the voluntary nature of the interviews. We used a case study design to evaluate TES, incorporating both quantitative (pre- and post-surveys) and qualitative (semi-structured interviews) approaches.

#### **Surveys**

We distributed pre- and post-surveys, administered at the beginning and end of each synchronous component, which was piloted in 2021. The pre- and post-surveys were designed to (1) evaluate the participants’ level of knowledge and confidence about the topics before and after the unit, (2) identify the reason for participating in the workshops, (3) capture the perceived benefits of prework, and (4) provide participants with the opportunity to share any comments about the pre-work.

#### **Interviews**

After completing TES, all participants were emailed an invitation to participate in a semi-structured interview. This email included information regarding the overall goals and motivation for doing the research. The interview questions (Supplemental Table 1) were designed to gain a deeper understanding of the participants’ reasons for attending the program, memorable units, what they applied from TES to their teaching, how the program has changed their view of teaching, any organizational impact, and future teaching directions for the participants.

We piloted the interview questions with 2 participants in our 2021 cohort, who were not included in this case study. A total of 15 participants (2022–2024) elected to participate in interviews, all conducted using Zoom. Interviews were facilitated by APG, DAH, and KMB and lasted around 1 h. All interviews were audio-recorded and transcribed verbatim, with the authors editing the transcripts during the first analysis phase. No repeat interviews were conducted.

The concept of “information power” guided the sample size determination [16]. Information power indicates that the more relevant information the sample holds for the study, the fewer participants are needed. After an initial analysis of the transcripts, we determined that sufficient data had been collected. This determination was based on several factors, including the high-quality dialogue between participants and interviewers, the specificity and purposiveness of the sampling, and the alignment of our interview data with the evaluation questions. Therefore, we did not seek additional interviews once it became clear that the new data yielded minimal novel information and that thematic sufficiency had been achieved.

### Data Analysis

#### **Statistical Analysis of Surveys**

Descriptive statistics were used to summarize the data. We used a pre-test/post-test design for each of the 5 Units to assess changes in knowledge and confidence. The exception was the Balancing Teaching, Research, and Service unit, for which a pre-test/post-test was not administered due to the unit’s design and content focus. Fisher’s Exact and Permutation Tests were used to analyze the difference in confidence levels; Wilcoxon Rank-Sum and Permutation Tests were used to analyze the difference in knowledge before and after each unit. Statistical significance was set at α = 0.05. Analyses were conducted using RStudio, Version 2025.5.1.513, Boston, MA.

#### **Thematic Analysis of Interviews**

We analyzed the textual data following Braun & Clark’s six-phase approach, including familiarization with the data, preliminary coding, theme identification, theme review, theme naming, data interpretation, and report production [17]. Two transcripts (from 2021) were used to create a preliminary codebook using inductive and deductive approaches. The codebook (Supplemental Figure) was then used to identify codes in the 15 additional transcripts (from 2022 to 2024). Transcripts were randomly assigned for coding by two of the three workshop presenters. Any coding differences were reconciled through discussion. Potential themes were reviewed throughout the coding process in a recursive manner. Analysis was conducted using Dedoose data analysis software (Version 9.2.5; SocioCultural Research Consultants, Los Angeles, California), until thematic sufficiency was achieved and themes were defined. The research team used iterative collaborative analysis to review, refine, and consolidate codes into themes that captured the complexity of participants’ experiences. The credibility of the findings was enhanced by investigator triangulation and continuous reflexive discussion, thereby reinforcing the findings’ reliability and trustworthiness. The Consolidated Criteria for Reporting Qualitative Research (COREQ) are included as a Supplemental Table 3 [18].

### Reflexivity & Trustworthiness

We assume that participants may conceptualize their teaching experiences differently based on their background and training. Therefore, we assembled a study team with diverse teaching and research backgrounds to ensure multiple perspectives in the collection, analysis, and interpretation of our data. Team members met regularly throughout the study and discussed our position regarding the data. APG and KMB are female basic science educators who had little to no formal pedagogical training during their graduate careers. APG was an LTF until 2020 and transitioned to a full-time teaching-focused faculty position in 2022. DAH is a clinician and began teaching in 2007. Prior to 2010, she had no formal pedagogical training. All individuals have terminal doctoral degrees (e.g., PhD, DHSc) and all completed the MTLDP. In 2021, TES was developed as APG’s final project in the MTLDP in collaboration with DAH and CFE. TB is a staff member and a former program associate of the CFE. She completed a Master’s degree in Human Resource Management in 2025. Our group included diverse research backgrounds. APG, KMB, and DAH have all had formal training at the master’s and/or doctoral level in quantitative and qualitative research. KMB and DAH have extensive experience in qualitative research, while APG is continuing to develop as a qualitative researcher, building on a strong foundation of formal training. TB’s research experience is primarily academic, through coursework at the undergraduate and master’s level that applied quantitative and qualitative methods.

We addressed the concept of trustworthiness through various strategies, including multiple-analyst triangulation of data, peer debriefing, and rich description. We considered using participant checks (i.e., transcript verification and interpretative verification); however, given the limited time of these volunteer study participants, we opted for peer debriefing, as we have a network of peers with formal training and experience in qualitative research available who were willing to support our efforts.

## Results

**Fig. 1:**
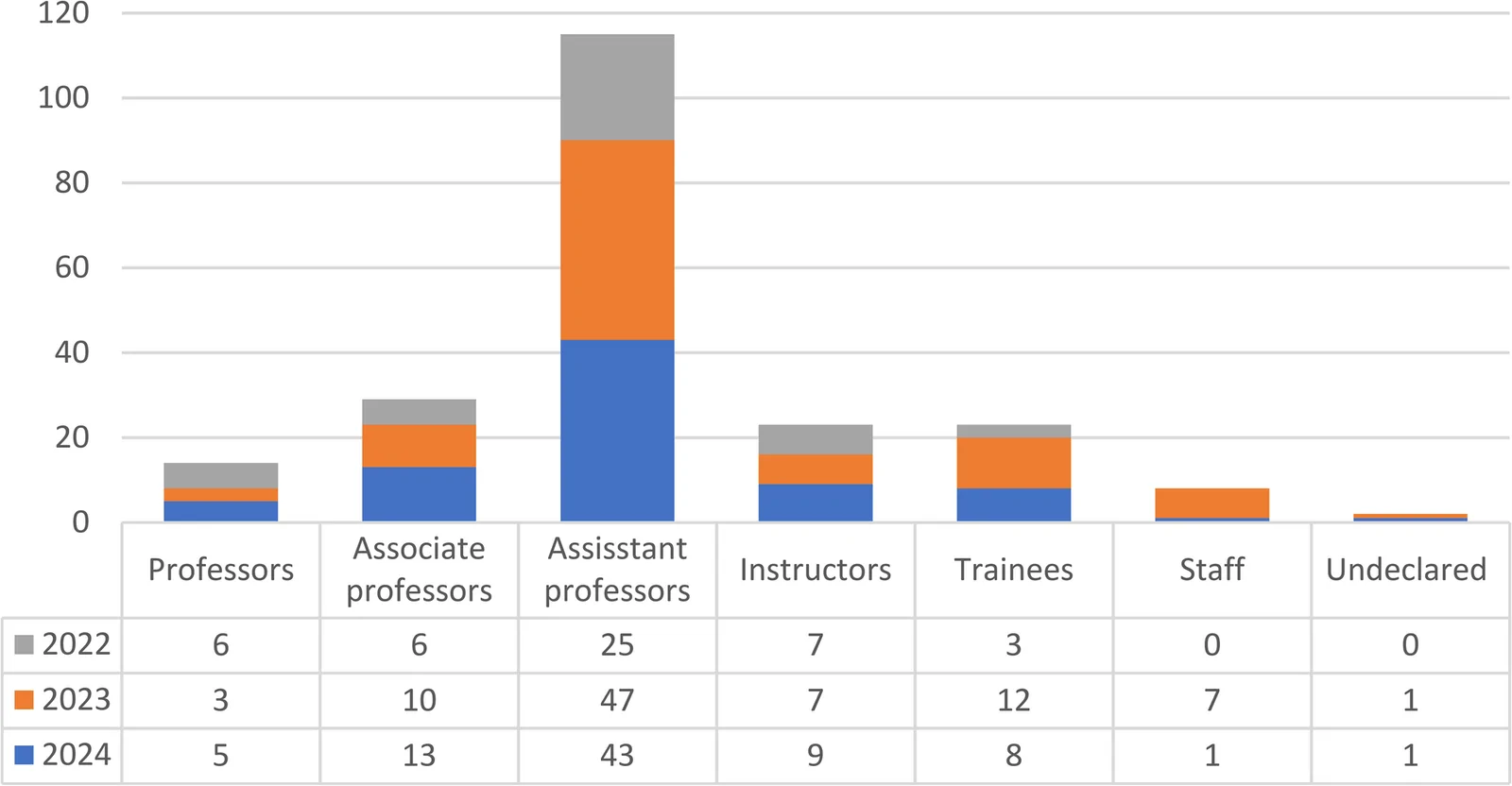
Study participants’ profile

Total participation for TES was 47 in 2022, 87 in 2023, and 80 in 2024 (*n* = 214). Participants included a spectrum of professionals in academic medicine, from staff to professors (Fig. 1), with early-career individuals (trainees, instructors, and assistant professors) representing the largest contingent (*n* = 161; 75.2%).

### Quantitative Data

#### Kirkpatrick’s Level 1-Reaction

This level measures participants’ immediate reactions to the training, including their satisfaction and engagement with the material. For this study, engagement with the material refers to whether participants completed the pre-work and found it beneficial. Satisfaction with the training was determined by the level of agreement with the following statements regarding the Units that: (a) the learning objectives were met, (b) the units were organized, and (c) the format and interactivity were effective. Regarding the pre-work, across all units, 50–71% of participants completed it, and up to 83% found it beneficial (Supplemental Table 4). Furthermore, participants reported high satisfaction after each unit (Table 1), with an overall mean score of 4.73 (SD = 0.55) on a 5-point Likert scale.

**Table 1:** Satisfaction likert scores after each unit likert mean (SD), 0–5 scale

Statements	Writing Learning Objectives (*n* = 44)	Assessment (*n* = 33)	Instructional Design (*n* = 22)	Writing MCQs (*n* = 15)	Balancing Teaching, Research, & Service (*n* = 16)	Overall (*n* = 130)
a) Learning objectives were met	4.75 (0.44)	4.91 (0.29)	4.82 (0.39)	4.73 (0.46)	4.38 (0.62)	4.73 (0.55)
b) Well-organized units	4.80 (0.41)	4.85 (0.44)	4.86 (0.35)	4.67 (0.49)	4.25 (0.86)	
c) Effective format & interactivity	4.70 (0.67)	4.79 (0.60)	4.82 (0.39)	4.67 (0.49)	4.25 (0.93)	

#### Kirkpatrick’s Level 2-Learning

This level assesses training effectiveness by measuring whether participants acquired the intended knowledge and whether their confidence in the topic improved. Wilcoxon-rank sum (W = 133.5; *p* = 0.007) and permutation (*p* = 0.010, Cohen’s *d* = 1.088) tests indicated that the correct rate after the Writing MCQs Unit was significantly higher, indicating that participants demonstrated a measurable gain in knowledge in writing MCQs. In contrast, the correct rates for other units did not differ significantly (Table 2).

**Table 2:** Mean correct rate and statistical tests of knowledge tests Pre- and Post-Unit

Unit Content (Sample Size)	Mean Correct Rate		Cohen’s d	Wilcoxon Rank-Sum Test Statistics W & *p*-value	Permutation Test *p*-value
	Pre-Session	Post-Session			
Writing Learning Objectives (*n* = 79)	0.703	0.759	0.396	936.5 & 0.050	0.127
Assessment (*n* = 87)	0.932	0.960	0.181	957& 0.335	0.483
Instructional Design (*n* = 37)	0.800	0.844	0.229	311.5 & 0.638	0.541
Writing MCQs (*n* = 26)	0.405	0.694	1.088	133.5 & 0.007*	0.010*

Note: Permutation tests were conducted with 5,000 resamples

An asterisk (*) denotes statistical significance at the 5% level

Confidence increased across all participants and units when comparing pre- and post-unit data; all participants reported at least feeling ‘Somewhat confident’. Fisher’s exact tests and permutation tests indicated that Likert mean scores for confidence levels were significantly higher after the Writing Learning Objectives (*p* < 0.0001; *p =* 0.0; Cohen’s *d* = 1.26), Assessment (*p* < 0.0001; *p* = 0.0; Cohen’s *d* = 1.53), and Instructional Design (*p* = 0.0498; *p* = 0.0262; Cohen’s *d* = 0.76) units (Table 3).

**Table 3:** Likert mean scores and statistical tests of confidence levels Pre- and Post-Unit

Unit Content (Sample Size)	Likert Mean (SD)		Maximum Likert Score	Cohen’s d	Fisher’s Exact Test *p*-value	Permutation Test *p*-value
	Pre-Session	Post-Session				
Writing Learning Objectives (*n* = 90)	2.52 (0.94)	3.48 (0.51)	4	1.26	< 0.0001 *	0 *
Assessment (*n* = 87)	2.02 (0.74)	2.94 (0.24)	3	1.53	< 0.0001 *	0 *
Instructional Design (*n* = 43)	2.19 (0.68)	2.64 (0.49)	3	0.76	0.0498 *	0.0262 *
Writing MCQs (*n* = 29)	2.21 (0.58)	2.6 (0.51)	3	0.72	0.1394	0.0940
Balancing Teaching, Research, and Service Strategies (*n* = 21)	1.9 (0.57)	2.45 (0.52)	3	1.01	0.1048	0.0620
Balancing Teaching, Research, Service Connections (*n* = 21)	1.8 (0.42)	2.36 (0.48)	3	1.24	0.0619	0.0312 *

Note: Permutation tests were conducted with 5,000 resamples

An asterisk (*) denotes statistical significance at the 5% level

Within the Balancing, Teaching, Research, and Service unit, confidence in applying strategies did not differ significantly before and after the unit. In contrast, confidence in connecting teaching, research, and service increased significantly, as indicated by the permutation test (*p* = 0.0312; Cohen’s *d* = 1.24). However, Fisher’s exact test did not detect a significant difference.

### Qualitative Data

We interviewed 15 participants representing a range of academic ranks (4 associate professors, 8 assistant professors, 2 instructors, and 1 trainee) across departments in the basic, health, and clinical sciences. Participants’ responses reflected all four levels of Kirkpatrick’s model. However, the relative dominance of the discourse at each level varied with the interview questions. The analysis identified four central themes.

#### Theme 1: Need for Formal Pedagogical Training

Participants highlighted a lack of formal training for teaching and expressed a strong desire for structured pedagogical training. Many noted that while they possessed deep content expertise, they had limited opportunities to develop foundational teaching skills. They viewed formal training as essential for improving effectiveness and enhancing learner engagement.

This theme was primarily observed in levels 1 and 2 of Kirkpatrick’s framework. The spectrum of prior training for teaching ranged from none at all to more structured, formalized training that provided insufficient detail or could not be applied immediately and was therefore forgotten. One participant (P) said, “*So I’m thinking of going into academia and becoming a research professor*,* and I had never had any formal training [in teaching]*” (P02, Kirkpatrick’s Level 1-Reaction).

Participants stated that they felt TES provided “*more tangible*” takeaways (P12, Kirkpatrick’s Level 1-Reaction). Some participants emphasized that although they received some formal training, the lack of opportunities to apply those skills made the training feel ineffective, “*I got some education [training on how to teach] that way. But if you don’t use it*,* like while I was in fellowship… it’s kind of useless*” (P15). They further expressed that TES offered them pedagogical training (Kirkpatrick’s Level 2-Learning), which helped them to become better educators, “*I’ve been looking for opportunities to gain additional formalized training to position myself to serve the students better*” (P05).

#### Theme 2: Value of Practical Learning, Shared Strategies, and Peer Connections

TES participants valued the practical knowledge gained and the shared tools and strategies rather than abstract educational theory alone. They emphasized actionable approaches and concrete examples that they could integrate into their own teaching contexts. Furthermore, they highlighted the opportunity to connect with and learn from peers they might not otherwise have met.

The benefits gained spanned across levels 1 through 3 of Kirkpatrick’s program evaluation framework. Participants reacted positively (Kirkpatrick’s Level 1-Reaction) to the program’s structure, which facilitated interaction with individuals across the academic medical enterprise and with the program overall. Although participants may have been at the same location (e.g., the main campus or an affiliate site), they did not necessarily have the opportunity to meet colleagues outside their immediate division or department. TES provided them with wider networks and communities for teaching practices,

*How else am I gonna get that exposure to different people from different disciplines working at [affiliate location]? [TES] provides an opportunity for the sharing of best practices*,* but across a wider group of faculty. So*,* I like that.* (P14)

Participants learned (Kirkpatrick’s level 2-Learning) the importance of fundamental teaching practices, such as aligning learning objectives, assessment methods, and teaching strategies, as well as writing effective MCQs. P15 explains,

*I think the main thing that really stuck out to me was [to] make sure that what you teach matches your objectives*,* and make sure that what you assess matches your objectives*,* and that seems really simple. But it’s something that I didn’t consciously put together before [TES].*

Participants found it beneficial that the knowledge and skills gained in TES could be applied (Kirkpatrick’s Level 3- Behavior) immediately or in future teaching, and it would help them “*get students more engaged*” (P08). P02 commented,

*I think if I [hadn’t been] teaching anything this semester*,* I probably wouldn’t have gotten as much out of it. It was because I had something that I could directly apply the concepts to … I hadn’t had any training before*,* so it was nice to just have some background reading before starting up on something that was new to me.*

#### Theme 3: Enhanced Confidence and Educational Profile

TES had a significant positive impact on participants’ confidence, enabling them to take ownership of their course materials, to aspire to greater success in their overall careers, and to strengthen their identities as educators in academic medicine.

Participants indicated that TES successfully achieved one of its primary goals: providing educators with limited prior training, the time and space to learn foundational teaching concepts. They discussed how critical the program was to addressing fundamental principles of effective teaching (Kirkpatrick’s Level 1-Reaction). Regardless of their prior teaching experience, most participants cited a desire to improve their teaching skills or become more effective educators as their primary motivation for registering for the units. P06 shared, “*I love teaching*,* and we can always grow*,* and I want to refine the skills to be more effective*” (Kirkpatrick’s Level 1-Reaction).

Participants’ confidence was predominantly displayed in Levels 1, 2, and 3 of Kirkpatrick’s evaluation framework. TES specifically increased participants’ confidence and knowledge (Kirkpatrick’s Levels 1 & 2) in developing instructional materials, thereby validating their decision-making.

*I think it also gave me a little bit of a boost in my confidence when I talk about these specific items*,* like objective writing. It also gives me a boost*,* when I’m thinking of evaluating the data that I received from a particular assessment*,* and whether or not…the [MCQ] item was appropriately written…*,* and whether or not throwing out the question is justified… I think it’s just essentially what it comes down to*,* just kind of boost of confidence that things that I was doing before are working*,* and I just gained more perspective as to why*,* because I got those feelings validated.* (P01)

Participants described how their participation in TES helped them gain confidence in other areas adjacent to teaching, such as pursuing new leadership roles in medical education or scholarship (Kirkpatrick’s Levels 1 & 3).

*As I’m kind of getting into my mid-career*,* from junior to mid-career faculty*,* I knew that education and teaching*,* I wanted that to be my main pillar. I saw that well*,* okay*,* I won’t be publishing much. But now*,* realizing that there actually is an opportunity for scholarship [in teaching]*,* I’m excited about that because I’ve done a lot of [non-education related] research and I don’t love it. It’s hard to do it when you don’t love it. But education is something I love.* (P07)

#### Theme 4: Shift in Teaching Perspective and Mindset

TES participants reported a shift in how they conceptualized and approached teaching following participation in the program. Rather than viewing teaching as primarily a content-delivery task, they adopted a more reflective, intentional, and collaborative approach. P01 said, “*this was an extremely helpful course for me because it’s kind of systematically [taught] me how to prepare my lectures and my practical activity*,* how to think through the long sections*,* how to do a lot of small things…”*.

Participants also reported applying newly acquired strategies beyond individual teaching roles. They shared practices with colleagues, collaborating on curriculum design and integrating insights from peers, demonstrating a more flexible and collaborative teaching mindset across departments, as evidenced in this quote from P06: “*I’ve been trying to be thoughtful of how to communicate some of this information to my team. So that way*,* [colleagues] can update their curricula and objectives*” (Kirkpatrick’s level 4-Results). Moreover, participants described sharing the strategies and insights they gained from the program with department leaders. By disseminating these practices upward and across the department, they created a ripple effect that extended the program’s impact beyond their own teaching. P13 stated,

*I just worked with some other faculty on rewriting learning objectives*,* and instructional objectives*,* and learning outcomes. And you know*,* working with our director of didactic education on curriculum mapping and also working with some other faculty*,* and developed kind of assessment guidelines for our faculty in terms of writing questions. So you know*,* taking what I’ve learned and expanded upon that*,* and to the other faculty.*

## Discussion

### Interpretation of Outcomes by Kirkpatrick Levels

Using Kirkpatrick’s evaluation framework [15], our findings indicate that participants viewed TES as beneficial. Confirmed by qualitative data, the quantitative data showed high satisfaction and engagement. The qualitative data further demonstrate that participants valued the interaction and social connectedness with peers gained from participation in TES (Kirkpatrick’s Level 1).

While knowledge (Kirkpatrick’s Level 2) of the TES topics was evident in the pre- and post-surveys, only one unit (Writing MCQs) demonstrated a statistically significant knowledge gain. For the other TES units, we believe participants may have been aware of the key concepts being assessed in the pre- and post-surveys but unsure how to apply them, as there is often a gap between knowing something and doing it.

Improvement in confidence (Kirkpatrick’s Level 2) was evident in all TES units, with the most significant increase in the Assessment unit, where confidence rose from 30% to 90%. The increase in confidence was also reflected in participants’ desire to pursue more educational opportunities and leadership roles, demonstrating the potential impact on their personal development beyond teaching and learning. These findings are consistent with similar studies on faculty development training programs for clinicians and basic science faculty [9, 19]. It is also important to acknowledge that self-reported confidence measures may be influenced by learners becoming more aware of gaps in their knowledge as they engage with new material, which in some cases could result in decreased confidence despite learning gains. However, this pattern was not observed in our findings, as both the quantitative survey data and qualitative interview responses consistently reflected increased confidence following participation in TES.

Unlike the vast majority of faculty development training program evaluation studies that only show evidence of Level 1 & 2 of Kirkpatrick’s framework [9, 19, 20], our data provides evidence that our participants changed how they teach and how they think about teaching (Kirkpatrick’s level 3) after participating in TES. For some of the study participants, these changes resulted in sharing of their new knowledge, skills, or ways of thinking about teaching with colleagues both within and across their divisions or departments, as well as across our academic enterprise, thereby suggesting that TES has the potential for organizational impact (Kirkpatrick’s Level 4). Our data did not demonstrate a clear organizational impact, likely because we did not design TES to include longitudinal participant engagement that would enable the collection and analysis of peer-reviewed teaching and student-teaching evaluation data.

### Contribution to the Literature and Field Significance

Our findings contribute to ongoing efforts to broaden access to effective teaching development by demonstrating that concise, practice-oriented programming can yield measurable learning and application outcomes. While many existing faculty development initiatives previously described [10–12], prioritize faculty with primary teaching roles and are more longitudinal, requiring substantial time, TES is intentionally designed for LTF educators with limited teaching loads and time available for training using pragmatic, immediately deployable strategies.

Key findings indicate that less time-intensive training can be effective. When program design includes clear learning objectives, active practice, and alignment with actual instructional challenges, faculty show increased learning, confidence, and the ability to apply and share new skills. Furthermore, although it is often difficult to demonstrate evidence at Kirkpatrick Levels 3 and 4 due to attribution and timing challenges, our study found that some participants self-reported implementing new strategies and sharing them with colleagues outside the program. This supports the view that focused, efficient training can promote the transfer of learning into practice, with the potential to positively impact the organization.

Although not part of our original design, the success of TES can also be explained by its alignment with the 4-Cs framework, first conceptualized by van Bruggen and colleagues and later endorsed by Lindholm and colleagues as a framework for mitigating the challenges and barriers to participating in faculty development programs [21, 22] The 4-Cs framework views faculty development programs across 4 domains: *Competence*,* Career*,* Context*, and *Community *[21]. The TES curriculum focused on 5 key topics, representing the *Competence* domain of the 4-Cs framework, which refers to the development of meaningful teaching skills that are necessary for participants to meet their teaching demands [21]. This focus on foundational skills provided all participants with an accessible starting point, one of the reasons our study found participants joined TES, regardless of prior experience.

Some participants sought to grow as educational leaders and scholars—a goal supported by the unit on Balancing Teaching, Research, and Service. This finding aligns with the shift in faculty development design toward not only skill acquisition but also leveraging participants’ motivation for personal and professional growth [19], reflecting the *Career* domain of the 4-Cs framework [21], which highlights that faculty development programs should ensure the career viability and vitality of faculty by providing opportunities for professional growth [22].

**Fig. 2:**
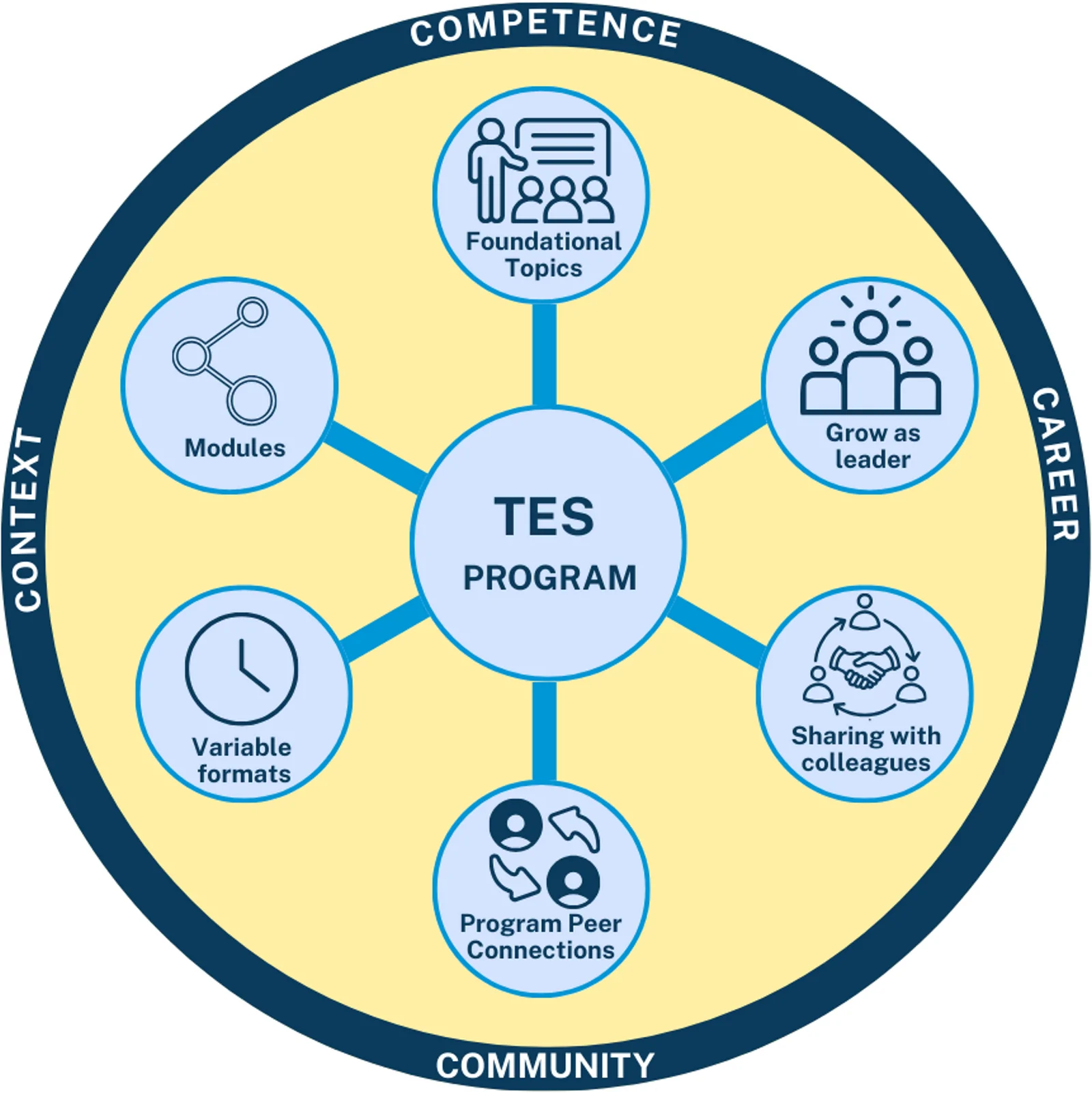
Conceptual model of how TES aligns with the 4-Cs Framework from van Bruggen et al., (2020)

The program’s modular, online units, combined with a time-efficient format, addressed common structural barriers such as limited availability and variable schedules, thereby addressing the *Context* domain, and acknowledging the time constraints and institutional realities that shape faculty participation [21]. The online synchronous format of the program also allowed for the unanticipated benefit of establishing peer connections and sharing ideas across various departments/divisions. This suggests that faculty development programs with a design similar to TES may serve as catalysts for the development of communities of practice focused on teaching excellence, thereby supporting the *Community* domain in the 4-Cs framework [21]. Steinart and colleagues purported that faculty members often learn about teaching through community participation and that “belonging to a community can enhance a sense of membership, promote transfer of training, and foster the attainment of individual goals” [19]. These researchers also suggest that the outcomes of these communities of practice can lead to cultural change, with institutional benefits through collective learning, the development of new knowledge, the acquisition of new skills, and improved practice [19] Fig. 2 summarizes the TES design elements within the broader 4-Cs framework [21].

Furthermore, it is noteworthy that some outcomes appeared to be influenced by participants’ professional backgrounds and teaching contexts. For example, research scientists highlighted increased confidence in curricular design and in developing learner-centered activities, whereas clinicians were more interested in the immediate applicability of strategies related to small-group teaching. Participants with prior teaching experience tended to describe TES as validating, while those with more limited formal training in education reported transformative changes in their teaching approach.

Taken together, the 4-Cs framework complements the Kirkpatrick framework by highlighting the mechanism by which TES outcomes are achieved. The 4-Cs framework helps explain how TES supports development, while the Kirkpatrick model provides a structure for categorizing the outcomes.

### Limitations and Future Direction

While our findings highlight several strengths of TES, they also reveal several limitations. For instance, many of our outcomes were based on participant self-reports, which may be subject to recall bias. Additionally, Kirkpatrick Levels 3 and 4 rely solely on evidence from those self-reports, as opposed to broader evidence from quantitative data like Levels 1 and 2. Furthermore, because of limited time commitment with participants (a strength of our training design), sustained behavior changes and institutional outcomes could not be fully measured. Additionally, because participants in TES teach only a small number of sessions, it was challenging to assess program impact using student or peer evaluations of teaching. While TES was intentionally designed as a relatively low-time-commitment faculty development model (5 h of synchronous components and 4 hours of asynchronous components for pre-work/post- work), this study did not directly compare participant workload, time investment, or outcomes with those of other faculty development approaches. Future studies should examine TES alongside alternative training models to better evaluate relative feasibility, effectiveness, and sustainability across institutional contexts.

While not one of the main research questions or themes identified, participants also discussed future enhancements that would improve TES (e.g., peer observation). Therefore, future iterations of TES will incorporate additional measures of teaching effectiveness, along with an assessment of student learning, as previously proposed [8]. We also hope to offer more structured, intentional opportunities to sustain cross-departmental collaboration and to share teaching practices beyond the program, through the development of communities of practice. Within these communities of practice, there will be an opportunity to collect and analyze longitudinal data to objectively measure personal and organizational impact. Lastly, we believe our results are transferable to other institutions and plan to disseminate the TES materials, tools, and associated activities to support the development of similar programs within their organizations.

## Conclusion

In this case study, we evaluated the TES program designed to equip research-intensive and clinician faculty with foundational teaching skills. Using Kirkpatrick’s evaluation framework as an organizing lens, we observed consistently high satisfaction and engagement (Kirkpatrick’s Level 1), measurable gains in teaching knowledge/skills and confidence (Kirkpatrick’s Level 2), and the application of core strategies through self-reported implementation in instructional settings (Kirkpatrick’s Level 3), with the potential for organizational impact (Kirkpatrick’s Level 4). We also demonstrate that TES aligns with the 4-Cs framework recommended for faculty development. Taken together, these findings suggest that a short, targeted intervention can support meaningful growth in essential teaching skills among research-intensive and clinician faculty who rarely teach and have limited time for training.

## Supplementary Information

Below is the link to the electronic supplementary material.

Supplementary Material 1 (DOCX 9.46 KB)

Supplementary Material 2 (DOCX 48.1 KB)

## Declarations

### Use of AI-Assisted Technologies

Generative AI (ChatGPT) and visual design and publishing tools (Canva) were used to improve language clarity and readability, and create models for this manuscript. To ensure scientific integrity and accuracy, all content was developed and authored by the listed authors, with any AI-generated suggestions carefully reviewed, checked for accuracy, and edited by all authors.
