# Supplementary material for: From Knowledge to Practice: Evaluating a Targeted Faculty Development Program for Medical Educators Using the Kirkpatrick Framework
Source: Med Sci Educ. 2026 Jul 9;36(5):2369–79. doi: 10.1007/s40670-026-02803-7 (PMC13723454; doi:10.1007/s40670-026-02803-7)
Supplement: Supplementary file 2 [file 40670_2026_2803_MOESM2_ESM.docx]

**Supplemental Table 1: Alignment of interview questions with the Kirkpatrick framework**

| **Question Type** | **Probes** | **Kirkpatrick framework levels** |
| --- | --- | --- |
| **Introductory** | Tell me about your teaching experience before the Teaching Essentials Series. | N/A |
|  | What attracted you to register for TES? | Reaction |
|  | Can you tell me more about any particular session you remember? | Reaction |
|  | Why do you remember this particular session? | Reaction |
|  | Why do you remember the particular session(s) better than others? | Reaction  Learning |
|  | What ideas, insights, solutions, or methods came out of it? | Learning |
| **Application of knowledge to teaching** | Have you applied anything from the TES to your teaching? | Learning  Behavior |
|  | If yes, describe what you implemented. | Learning  Behavior |
|  | If not, why not (what barriers/challenges did you face)? | Learning  Behavior |
| **Personal transformative value** | Do you think the TES program has changed how you feel about teaching? | Learning  Behavior |
|  | Has it changed how you see yourself? How? | Learning  Behavior |
| **Organizational transformative value** | What, if any, impact has the TES program had on your work? (With students, colleagues, faculty, staff, etc.) | Results |
|  | Have you observed any organizational changes (department, program, team, etc.) due to your participation in the TES series? | Results |
| **Aspirations** | What additional aspirations do you have now? | Behavior  Results |
|  | How have these changed since the completion of the program? | Learning  Behavior  Results |
| **Closing questions** | What (if anything) should we discuss that was not covered? | N/A |
|  | What other sessions, if any, would you like to see? | N/A |
|  | Is there anything else you want to share about your experience in the TES program and how to be an educator? | N/A |

**Supplemental Table 2: Overview of TES program topics, learning objectives, and activities**

| **Topics** | **Module Description** | **Learning Objectives of Each Unit** | **Activities for Each Component** |
| --- | --- | --- | --- |
| **Writing Learning Objectives** | This module will introduce participants to Learning Objectives and will provide context for why we need them and how to write them. In this module, participants will practice writing and refining learning objectives. Peers and instructors will provide feedback on learning objectives written or revised. | 1. Identify key components of a well-written learning objective  2. Discuss the process for writing learning objectives  3. Practice writing effective learning objectives | *Asynchronous components*  1. Watch the learning objective video (10 minutes)  2. Draft 3 of your learning objectives (15 minutes)  *Synchronous components*  3. Attend the Zoom session (1 hour)  4. Complete the pre-session survey (5 minutes)  5. Complete the post-session survey (5 minutes) |
| **Assessment** | This module will introduce participants to formative, summative, and classroom assessments of learning. The importance of aligning assessments with learning objectives/outcomes will be discussed. | 1. Describe characteristics of formative and summative assessments  2. Explain curricular alignment between assessments, learning objectives, and teaching strategies  3. Relate an assessment to a given learning objective | *Asynchronous components*  1. Watch the assessment video (10 minutes)  2. Draft 1-2 assessments that map to the learning objective for a session you teach (10 minutes)  *Synchronous components*  3. Attend the Zoom session (1 hour)  4. Complete the pre-session survey (5 minutes)  5. Complete the post-session survey (5 minutes) |
| **Instructional Design** | This module will introduce participants to the **PHLET** format for designing instruction. When designing instruction, educators need to **P**repare and **H**ook the learner, **L**ink to their prior knowledge/experiences, **E**ngage them in the learning process, and help them **T**ransfer their knowledge to new or novel experiences. | 1. Identify the major components of effective instructional design  2. Refine the design of a teaching session to reflect the required components | *Asynchronous components*  1. Watch the session design video (15 minutes)  2. Draft pre-link-hook elements for a session you teach & submit as the PHLET assignment (15 minutes)  *Synchronous components*  3. Attend the Zoom session (1 hour)  4. Complete the pre-session survey (5 minutes)  5. Complete the post-session survey (5 minutes) |
| **Writing Multiple-Choice Questions** | This module will introduce participants to writing high-quality, board-style multiple-choice questions to assess learning. | 1. Recognize the most common errors in the components of written exam questions  2. Apply best practices in the construction of MCQs for examination | *Asynchronous components*  1. Watch the NBME Item Writing Tutorial (45 minutes)  *Synchronous components*  2. Attend the Zoom session (1 hour)  3. Complete the pre-session survey (5 minutes)  4. Complete the post-session survey (5 minutes) |
| **Balancing Teaching, Research & Service** | This module will provide participants with tips and perspectives to help them balance teaching, research, and service responsibilities. | 1. Identify ways to balance teaching, research, and service in one’s career  2. Describe ways one's research, teaching, and service can be connected  3. Recognize resources and supports available to health professions education academics | *Asynchronous components*  1. Complete the pre-session survey (5 minutes)  2. Vote on pre-selected questions for panelists (5 minutes)  *Synchronous components*  3. Attend the Zoom session/Panel format with Q & A (1 hour)  4. Complete the post-session survey (5 minutes) |

**Supplemental Table 3: Consolidated criteria for reporting qualitative studies (COREQ)**

| **No. Item** | **Guide questions/description** | **Reported on Page #** |
| --- | --- | --- |
| **Domain 1: Research team and reﬂexivity** |  |  |
| ***Personal Characteristics*** |  |  |
| 1. Inter viewer/facilitator | Which author/s conducted the interview or focus group? | 6 |
| 2. Credentials | What were the researcher’s credentials? (E.g. PhD, MD) | 8 |
| 3. Occupation | What was their occupation at the time of the study? | 8 |
| 4. Gender | Was the researcher male or female? | 8 |
| 5. Experience and training | What experience or training did the researcher have? | 8 |
| ***Relationship with participants*** |  |  |
| 6. Relationship established | *Was a relationship established prior to study commencement?* | 6 |
| 7. Participant knowledge of the interviewer | *What did the participants know about the researcher? (e.g. personal goals, reasons for doing the research).* | 6 |
| 8. Interviewer characteristics | *What characteristics were reported about the interviewer/facilitator? (e.g. Bias, assumptions, reasons and interests in the research topic)* | 8 |
| **Domain 2: Study design** |  |  |
| ***Theoretical framework*** |  |  |
| 9. Methodological orientation and Theory | *What methodological orientation was stated to underpin the study? (e.g. grounded theory, discourse analysis, ethnography, phenomenology, content analysis).* | 4 |
| ***Participant selection*** |  |  |
| 10. Sampling | *How were participants selected? (e.g. purposive, convenience, consecutive, snowball)* | 6 |
| 11. Method of approach | *How were participants approached? (e.g. face-to-face, telephone, mail, email)* | 6 |
| 12. Sample size | *How many participants were in the study?* | 6, 8 |
| 13. Non-participation | *How many people refused to participate or dropped out? Reasons?* | 6, 8 |
| ***Setting*** |  |  |
| 14. Setting of data collection | *Where was the data collected? (e.g. home, clinic, workplace)* | 6 |
| 15. Presence of non-participants | *Was anyone else present besides the participants and researchers?* | 6 |
| 16. Description of sample | *What are the important characteristics of the sample? (e.g. demographic data, date)* | 8 |
| ***Data collection*** |  |  |
| 17. Interview guide | *Were questions, prompts, guides provided by the authors? Was it pilot tested?* | 7 |
| 18. Repeat interviews | *Were repeat interviews carried out? If yes, how many?* | 7 |
| 19. Audio/visual recording | *Did the research use audio or visual recording to collect the data?* | 7 |
| 20. Field notes | *Were ﬁeld notes made during and/or after the interview or focus group?* | No |
| 21. Duration | *What was the duration of the inter views or focus group?* | 7 |
| 22. Data saturation | Was data saturation discussed? | 7 |
| 23. Transcripts returned | *Were transcripts returned to participants for comment and/or correction?* | 8 |
| **Domain 3: analysis and ﬁndings** |  |  |
| ***Data analysis*** |  |  |
| 24. Number of data coders | How many data coders coded the data? | 7 |
| 25. Description of the coding tree | *Did authors provide a description of the coding tree?* | Supplemental Figure |
| 26. Derivation of themes | *Were themes identiﬁed in advance or derived from the data?* | 7 |
| 27. Software | *What software, if applicable, was used to manage the data?* | 7 |
| 28. Participant checking | *Did participants provide feedback on the ﬁndings?* | 8 |
| ***Reporting*** |  |  |
| 29. Quotations presented | *Were participant quotations presented to illustrate the themes/ﬁndings? Was each quotation identiﬁed? (e.g. participant number)* | 9-13 |
| 30. Data and ﬁndings consistent | *Was there consistency between the data presented and the ﬁndings?* | 9-13 |
| 31. Clarity of major themes | *Were major themes clearly presented in the ﬁndings?* | 9-13 |
| 32. Clarity of minor themes | Is there a description of diverse cases or discussion of minor themes? | 16 |

**Supplemental Table 4: Pre-Work Engagement**

| **Survey questions** | **Writing Learning Objectives** | **Assessments** | **Instructional Design** | **Writing MCQs** | **Balancing Teaching, Research, and Service** |
| --- | --- | --- | --- | --- | --- |
| **Completed pre-work (%)** | 71 | 65 | 67 | 54 | 50 |
| **Found it beneficial (%)** | 83 | 78 | 73 | 77 | 50 |
