# Supplementary material for: From Knowledge to Practice: Evaluating a Targeted Faculty Development Program for Medical Educators Using the Kirkpatrick Framework
Source: Med Sci Educ. 2026 Jul 9;36(5):2369–79. doi: 10.1007/s40670-026-02803-7 (PMC13723454; doi:10.1007/s40670-026-02803-7)
Supplement: Supplementary file 3 [file 40670_2026_2803_MOESM1_ESM.docx]

Supplemental Figure: Code Tree
